# Collecting and managing *in situ* banana genetic resources information (*Musa* spp.) using online resources and citizen science

**DOI:** 10.1093/database/baae036

**Published:** 2024-05-22

**Authors:** Christophe Jenny, Valentin Guignon, Felip Manyer I Ballester, Max Ruas, Mathieu Rouard

**Affiliations:** CIRAD, UMR AGAP Institut, University of Montpellier, F-34398, France; UMR AGAP Institut, University of Montpellier, CIRAD, INRAE, Institut Agro, Montpellier, F-34398, France; UMR AGAP Institut, University of Montpellier, CIRAD, INRAE, Institut Agro, Montpellier, F-34398, France; Bioversity International, Parc Scientifique Agropolis II, 34397, Montpellier, France; CIRAD, UMR AGAP Institut, University of Montpellier, F-34398, France; UMR AGAP Institut, University of Montpellier, CIRAD, INRAE, Institut Agro, Montpellier, F-34398, France; UMR AGAP Institut, University of Montpellier, CIRAD, INRAE, Institut Agro, Montpellier, F-34398, France; Bioversity International, Parc Scientifique Agropolis II, 34397, Montpellier, France; UMR AGAP Institut, University of Montpellier, CIRAD, INRAE, Institut Agro, Montpellier, F-34398, France; Bioversity International, Parc Scientifique Agropolis II, 34397, Montpellier, France

## Abstract

The Musa Germplasm Information System (MGIS) stands as a pivotal database for managing global banana genetic resources information. In our latest effort, we have expanded MGIS to incorporate *in situ* observations. We thus incorporated more than 3000 *in situ* observations from 133 countries primarily sourced from iNaturalist, GBIF, Flickr, Pl@ntNet, Google Street view and expert curation of the literature. This addition provides a more comprehensive and detailed view of banana diversity and its distribution. Additional graphical interfaces, supported by new Drupal modules, were developed, allowing users to compare banana accessions and explore them based on various filters including taxonomy and geographic location. The integrated maps present a unified view, showcasing both *in situ* observations and the collecting locations of accessions held in germplasm collections. This enhancement not only broadens the scope of MGIS but also promotes a collaborative and open approach in documenting banana diversity, to allow more effective conservation and use of banana germplasm. Furthermore, this work documents a citizen-science approach that could be relevant for other communities.

**Database URL**: https://www.crop-diversity.org/mgis/musa-in-situ

## Introduction

*In situ* conservation is crucial as it preserves rich species genetic diversity in natural habitats, allowing for ongoing evolution in response to environmental changes. In addition, it maintains sources of resilience to withstand challenges like climate change, ensuring sustainable use and future research opportunities (1, 2). Recent developments of digital tools like iNaturalist (3), GBIF (4) and Pl@ntNet (5) have enhanced *in situ* conservation efforts by allowing people to contribute in real-time to the monitoring and identification of existing diversity in their natural habitats. By using such platforms, any citizen, including researchers, can help identify and collect information, which may be exported and combined with complementary data for subsequent analyses such as gap analyses for crop conservation.

Bananas (*Musa* spp.) originate from Southeast Asia and Oceania and are cultivated worldwide across the tropics and subtropics. Most of the varieties grown in the world today are triploid plants derived from two main wild species, *Musa acuminata* and *Musa balbisiana* (6). In the mid-twentieth century, a morphotaxonomic characterization system was developed (7) to help recognize and identify the various varieties and natural hybrids cultivated today. These cultivated types are classified within botanical groups, most of which can be readily identified using these morphotaxonomic descriptions (8, 9). All other natural wild species are also described and may be identified by experts (10). This information can be used to identify varieties and cultivated types based on pictures, commonly displayed in dedicated online databases and photographic websites. Recording the geographical extension of *Musa* diversity has various purposes. Bananas grow in a wide range of areas, some of which may be threatened by climate change or urbanization. Knowing this distribution precisely helps identify current and future threats and propose alternative risk mitigation solutions. Wild types and natural biodiversity are also at risk. The preservation of these types is based on knowledge of their distribution. Protective collecting missions can also be planned in these areas to fill gaps in our *ex situ* collections, or *in situ* conservation may be justified based on this information. For instance, geneticists recently discovered that unknown ancestors contributed to the genetic makeup of cultivated bananas (11, 12), and they are still to be found.

Information on banana diversity for *ex situ* collections has been documented in the *Musa* Germplasm Information System (MGIS) (13) as a community effort (14), and the system provides curated taxonomy and maps for the visualization of germplasm collection as well as the location of collected material. With 7020 accessions managed in 32 germplasm collections around the world, as of October 2023, MGIS is the most extensive source of information on banana genetic resources. While an extensive *ex situ* dataset in MGIS provides critical insights into banana genetic resources, the inclusion of *in situ* observations offers a richer, more comprehensive perspective on the distribution of banana diversity. With this in mind, we harnessed the capabilities of specialized digital platforms to seamlessly integrate diverse content on Musaceae, contributed by a wide range of individuals—from scientists and researchers to the general public and banana enthusiasts.

The goal of this exercise has been to enrich MGIS with a significant amount of *in situ* observations, and to establish a continuous, dynamic process that would allow for the integration of these observations into MGIS. By doing so, we also intend to foster a cost-effective and diversified approach that builds on and values contributions from the wider community.

## Database content

### *In situ* observation data retrieval

**Figure 1.**
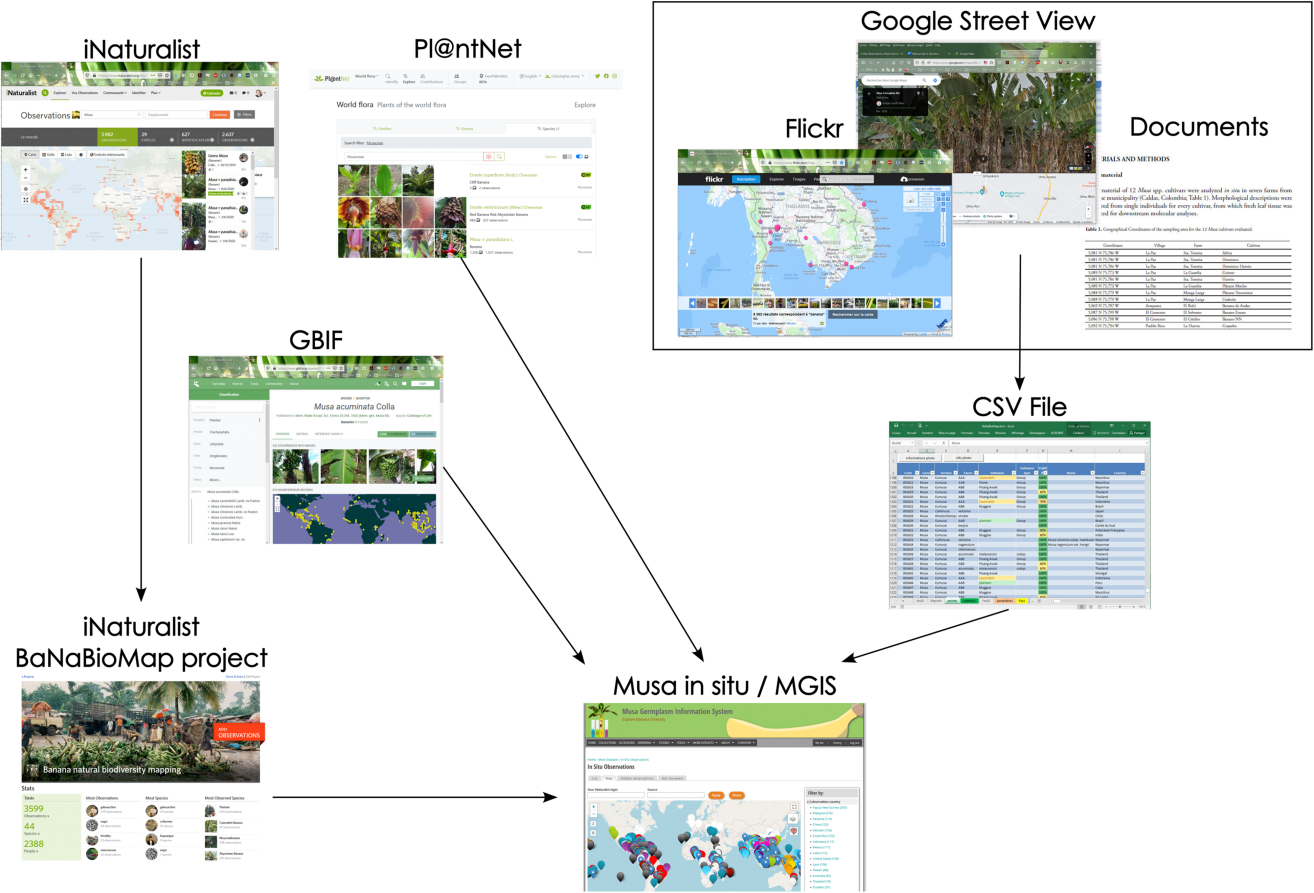
Schematic process of data retrieval from various data sources (e.g. iNaturalist, GBIF, Pl@ntNet, Flickr, Google Street View and Documents) toward curation and management in the MGIS.

As of November 2023, slightly more than 5000 observations from 133 countries were selected from multiple sources including the specialized digital platforms iNaturalist, GBIF and Pl@ntNet and general tools like Flickr and Google Street View and from collecting mission reports and scientific literature (Table 1). So far, 93 different taxa, encompassing both wild and cultivated varieties, have been identified within the three Musaceae genera, *Musa, Ensete* and *Musella*, and these are distributed across 133 countries. These observations were retrieved, curated and stored as illustrated in Figure 1.

**Table 1.** Number of observations per source as of November 2023

		Imported in MGIS^b^			
Data sources	Raw data in source^a^	Displayed	Not reviewed	Hidden	Waiting list^c^
iNaturalist (banabiomap project)	3646	2017	4	105	1520
Flickr	479	444	0	0	35
GBIF	637	632	0	5	0
Pl@ntNet	104	32	30	42	0
Google Street View	11	11	0	0	0
Literature	219	53	0	0	166
**Total**	**5096**	**3189**	**34**	**152**	**1721**

a Total number of observations identified for each source type.

b Observations already imported in MGIS, and their status. Observations displayed are public. Observations not reviewed are waiting for expert analysis. Observations marked as ‘hidden’ have been discarded from public display, generally because taxonomic identification is not available. Observations are kept in the database to avoid future importation again.

c The waiting list includes observations not yet imported from the source (BaNaBioMap project or CSV exchange file).

For a thorough understanding, detailed descriptions of each data source used are provided below:

### iNaturalist

iNaturalist (www.inaturalist.org) is an online social network of people sharing biodiversity information to help each other learn about nature. It is a crowdsourced, species-identification system and an organism-occurrence recording tool. In 2023, iNaturalist became an independent non-profit organization. Internationally, iNaturalist partners with several different organizations through the iNaturalist Network to provide a localized experience along with greater reach and impact.

A daily watch of observations classified as belonging to the Musaceae family has been performed since 2020. Observations are checked, and if identification is possible up to the cultivar group level or to the species/subspecies level for wild types, it is retained. To facilitate this process, we created BaNaBioMap (www.inaturalist.org/projects/banana-natural-biodiversity-mapping), a dedicated ‘project’ within the iNaturalist website to store these observations. This is a convenient way to store in the same place, selected observations. As of end of November 2023, a little more than 3600 observations have been included in the project, which represents roughly 10% of all observations classified as Musaceae in iNaturalist. iNaturalist offers the facility of adding personalized variables to any project. In BaNaBioMap, we defined mandatory descriptors to help refining plant identification and plant behavior description: (i) banana botanical classification (main cultivated groups), (ii) banana observation source (environment) and (iii) banana plant condition. To date, iNaturalist is by far the most important source of data for our project (Table 1). The website is steadily gaining in popularity, with more and more observations being posted month after month. New areas are regularly covered, and a network of passionate users is gradually being built up. The only drawback is that bias can occur, linked to uneven user participation from different countries, leading to unbalanced numbers of observations. We try to counterbalance this bias by varying the sources of our observations.

### GBIF portal

GBIF, the Global Biodiversity Information Facility (www.gbif.org), is an international biodiversity data network and infrastructure funded by governments worldwide. Its remit is to provide open access to information on all life on Earth derived from diverse sources such as historical museum specimens, recent DNA barcodes or smartphone photos. GBIF draws together these varied data through standards like Darwin Core (dwc.tdwg.org), which underpins the network’s indexing of hundreds of millions of species-occurrence records. GBIF presents a huge database, but most records must be considered ‘as is’, as the vast majority do not include photos that could help validate the information. No data selection is carried out by GBIF itself, which declares itself incompetent in this field. Records are therefore filtered mainly on the basis of the datasets’ authors’ names, choosing recognized experts whose botanical identification cannot be questioned.

To import GBIF observations into the MGIS database, we developed and released a dedicated Drupal module (www.drupal.org/project/gbif2). It is part of MGIS back office and provides a GBIF observation browser that can list and filter available observations to select and import the most appropriate ones. While its first version was designed for MGIS, this Drupal module is generic enough to be used by other websites with other taxa. It takes advantage of the concept of ‘external entities’ (www.drupal.org/project/external_entities), useful to map ‘external’ (in the sense of ‘not stored in Drupal database’) data as Drupal entities, which is the preferred way to model and work with data in Drupal. We believe that this concept is particularly relevant in this field of data management, and plan to elaborate on this in a forthcoming publication.

The GBIF Drupal module relies on the GPLv3 PHP-GBIF library (packagist.org/packages/restelae/php-gbif), which is a partial port (written in the context of this project) of the Python pygbif library (github.com/gbif/pygbif) (15). These libraries are clients for the GBIF REST Application Programming Interface (API) (www.gbif.org/developer/summary), which aim to ease the process of consuming and processing data from GBIF.

### Pl@ntNet

Pl@ntNet (plantnet.org) is a collaborative international platform that uses machine learning to identify plants from photographs. Through the mobile app and website, users can submit photos to be analyzed against Pl@ntNet’s database of hundreds of thousands of scientifically validated plant images. This allows anyone to identify thousands of plant species found globally. Since its launch in 2014, Pl@ntNet has facilitated nearly four million identifications for both professionals and the public across 22 projects spanning major bioregions. Governed by a consortium of research institutions (CIRAD, Inria, INRAE, IRD and the Agropolis Foundation), Pl@ntNet helps to build global plant biodiversity knowledge and enhance biodiversity conservation through easy plant identification via photography. Although primarily focused on identification, Pl@ntNet also hosts location data which may be released as public information.

For data retrieval, we used a manual JavaScript Object Notation (JSON) export of public observations of banana plants. The export file was processed and integrated into MGIS using a dedicated migration plan with the Drupal Migrate module. While we filtered observations with public geolocation (in areas of interest), Pl@ntNet data do not include the country codes of records. To enable filtering those observations by country, we relied on the Genesys web service (www.genesys-pgr.org/documentation/apis) to identify country codes from GPS coordinates. Most of the Pl@ntNet data import process is already automated and this will be fully completed in the next release of MGIS. This will be achieved in collaboration with the Pl@ntNet developers and will include the improvement of the Pl@ntNet API, offering an endpoint to access observations.

### Flickr

Searches were carried out on Flickr (www.flickr.com), a historical database offering quality online photographs of all kinds. Relevant observations were searched on the map page focused on areas of interest: www.flickr.com/map. The research toolbar located under the thumbnail’s gallery allows you to find pictures meeting specific keywords, present in the current view. When botanical identification was possible, metadata were retrieved using the *flickr.photos.getinfo* API (www.flickr.com/services/api/explore/flickr.photos.getInfo) and pasted in a local exchange file. Metadata include date of record, administrative location and geographic coordinates, along with an uncertainty value. Picture links for later illustration were only saved where the copyright license complied with public sharing (namely CC-BY licenses).

### Google Street View

**Figure 2.**
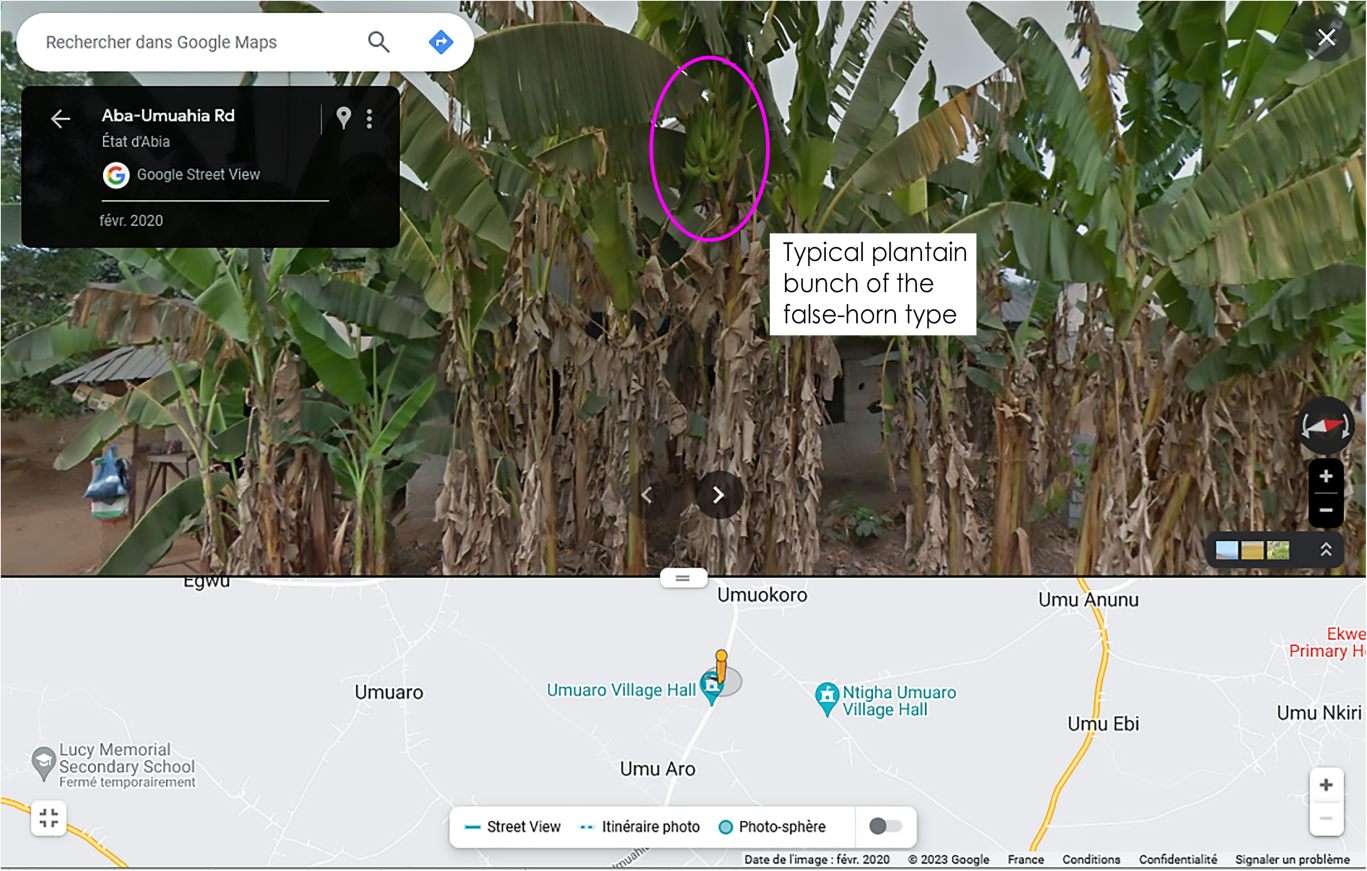
Spotting a plantain plant in a village in Ghana using Google Street View.

Google Street View is a feature integrated into both Google Maps and Google Earth, offering interactive panoramas from various road locations around the globe. It is possible to target a region of interest and explore views. For a picture, it is then possible to retrieve the geographic coordinates (included in the URL), the name of the location and the date. The quality of the pictures has often enough resolution for an expert to allow botanical identification for the main cultivated groups of bananas as exemplified in Figure 2. At present, Google Street View does not cover the whole Earth, but it is sometimes present in areas where leisure pictures are scarce and can usefully complement the geographical distribution of observations and help correct the sampling bias observed with iNaturalist-only observations. For instance, the resource has been tested in Nigeria and Ghana for Plantain types, and in Bangladesh for Pisang Awak and Cavendish banana types. Again, when identification was possible, metadata were manually retrieved and pasted in the local CSV exchange file.

### Scientific and technical literature

A wide diversity of interesting sources is not available online, or at least is not present in online databases. This is the case for scientific and technical literature that often hosts references to geolocated biological material. The list includes collecting mission reports and also botanical articles or research projects about new species or natural diversity in specific areas for instance. Personal blogs by amateur botanists or horticulturists are also often interesting. These publications often focus on the center of origin of the plants, and for bananas, they mostly take place in Southeast Asia (16–20). This is highly valuable information about the distribution area of wild types, ancestors of cultivated bananas and landraces. In this case, the main issue is data formatting, which often lack standardization to be consistently processed by automated scripts. It may be displayed in tables or included in plain text. Extracting information using generative AI models has been tested in both cases with free models such as OpenAI Chat-GPT 3.5 and Claude. Examples of prompts are provided as Supplementary data. All data have been double checked for consistency, but so far, no or very few errors have been identified. Even where corrections were made, using this process greatly accelerated data retrieval.

For publications published prior to GPS use, locations were typically inferred from administrative information, which may vary in precision. This was the case of historical herbaria for instance. This information remains interesting, as it may provide timestamps, witnessing the presence, disappearance or displacement of plants. AI models with access to the internet can also be used here, with limitations related to the precision of the available information. Examples of prompt and search results using Google PaLM and Solar by Upstage are given in Supplementary data.

### Expert curation process

Data collection necessitates essential curation to ensure its quality. Post-processing has been conducted within the back office of the MGIS website. Each observation imported into MGIS undergoes thorough analysis before it is made publicly available on the website.

This included:

- Definitive taxonomic treatment, using the imported data: banana taxonomy is relatively challenging, and related information is often not managed appropriately on all platforms. Accurate taxonomy must be applied, translating the original information into the MGIS Musa taxonomic scheme. Observations that do not allow identification are discarded.
- Location: GPS coordinates are imported, but the altitude of points is not provided, whatever the source of the data. Google Maps does not provide the altitude of points. However, various tools are available to find it, and some of them, such as Google Earth or CalcMaps (www.calcmaps.com/fr/map-elevation/), were used to retrieve missing data points.
- Licenses: when the picture license allows, photos of the plant are retrieved. This is typically the case for all CC-BY license types. If more than one picture is available, the choice of the featured individual is made in the back office. For pictures under © license, only a link to the original observation is kept. For observation data, we retrieved only those under CC-BY license types, excluding all right reserved.

To be recorded and included, an observation must meet the following requirements:

- Botanical classification of the plant must be possible up to the species or subspecies level (wild types), or to the cultivar group (cultivated types).
- The plant must be growing in as close to natural conditions as possible. An emphasis is put on plants that have not been cultivated at all. If cultivated, valid points are limited to ‘backyard’ plants, or small personal plantations without any intensive care.
- The geographical location must be as precise as possible and, in any case, approximately less than 1 km.

Observations which do not meet these requirements are tagged as hidden and not publicly displayed but kept in the database to avoid a future new import. These hidden observations may also be modified, and their status revised whenever the source data become complete.

Data redundancy is not a problem, and although species rarity is an important criterion, we also record the extent of distribution of popular types. The aim is to be able to map the distribution areas of the different banana types. We hence aim to (i) produce as complete a map as possible of classic cultivated banana types and (ii) map the occurrence and extent of rare types in their natural areas of distribution.

## System architecture

**Figure 3.**
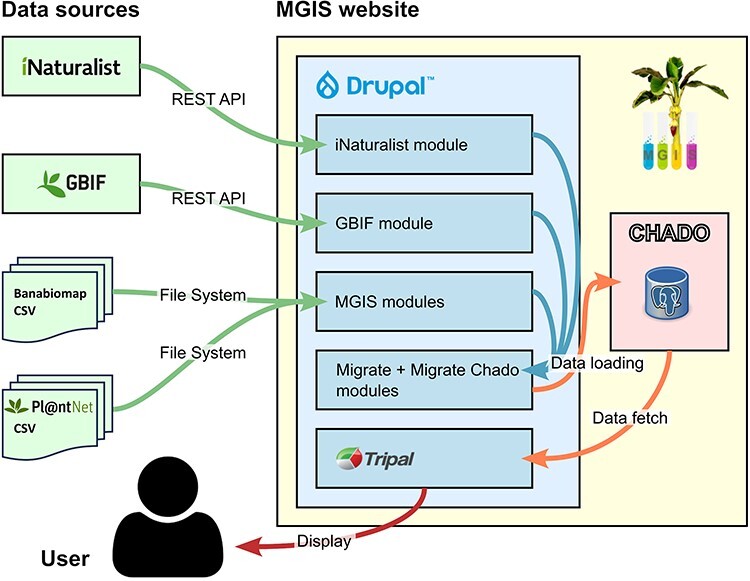
Observation data flow in the MGIS.

MGIS is implemented using the Drupal content management system (CMS) (www.drupal.org/) combined with the Tripal module (21,22), and is associated with the standard Chado database schema (23). As the objective has been to extend the MGIS database by adding *in situ* data, for data collection and synchronization, we implemented a combination of existing and tailor-made Drupal modules. Such modules were developed to directly import, from Drupal backend, data from iNaturalist (www.drupal.org/project/mgis_inaturalist) and GBIF portals (www.drupal.org/project/gbif2) using their respective APIs (Figure 3), enabling regular synchronization. We complemented it with an interface for importing *ad hoc* CSV files containing manually collected data, including Pl@ntNet, whose API does not yet allow such automated calls.

The Chado database’s inherently flexible structure allows for versatile management of identical datasets in various ways. In our case, *in situ* observations are efficiently cataloged as Chado stock records within the stock table. This is achieved using specific type identifiers in the stock.type_id column, which serve to distinguish between different sources of observations. GPS coordinates are recorded in the Natural Diversity Chado module (24) using a combination of the nd_experiment_stock, nd_experiment and nd_geolocation tables for precise geospatial data representation. Furthermore, external references, such as links to public observation webpages, or associated images, are systematically stored in the stock_dbxref table as stock cross-references. Additional attributes, including the source user, country of origin and various comments, are captured in the stockprop table. Similarly, controlled vocabulary terms related to the plant condition, observation source and other relevant categories are stored in the stock_cvterm table.

Data loading into the Chado database is performed with the help of the Migrate module ecosystem. Migrate is an ETL (extract, transform, load) framework for Drupal, which can be extended to extract data from various sources (e.g. from a REST API or a CSV file) or to load them into various destinations (for instance as Drupal entities or into a third-party database). Precisely, to that end, we extended the base module with a new one, called Migrate Chado, to enable Migrate to store collected data within the Chado schema instead of the Drupal schema, as we prefer to separate CMS data from biological data. The biological data stored in Chado schema remain accessible to Drupal through the Tripal module and MGIS custom modules.

We enhanced the existing MGIS *ex situ* data browsing interfaces by adding comparable *in situ* interfaces. To easily filter and list observations, they are indexed using Drupal Search API ElasticSearch module in combination with the Facet module. Matching observations are displayed either in a results table or on a map using a set of Drupal Views and OpenStreetMap modules. On the backend, we also developed an observation curation interface that allows reviewing and selecting observations and pictures to display and adjust their taxonomy.

## How to use the website

To accommodate and display the specificities of *in situ* data, we introduced a suite of new interfaces. These were designed to complement the existing ones in MGIS, ensuring coherent integration. From search capabilities to dynamic visualization tools, these interfaces aim to benefit from core information provided by external data sources and enrich it with data curation and integration.

**Figure 4.**
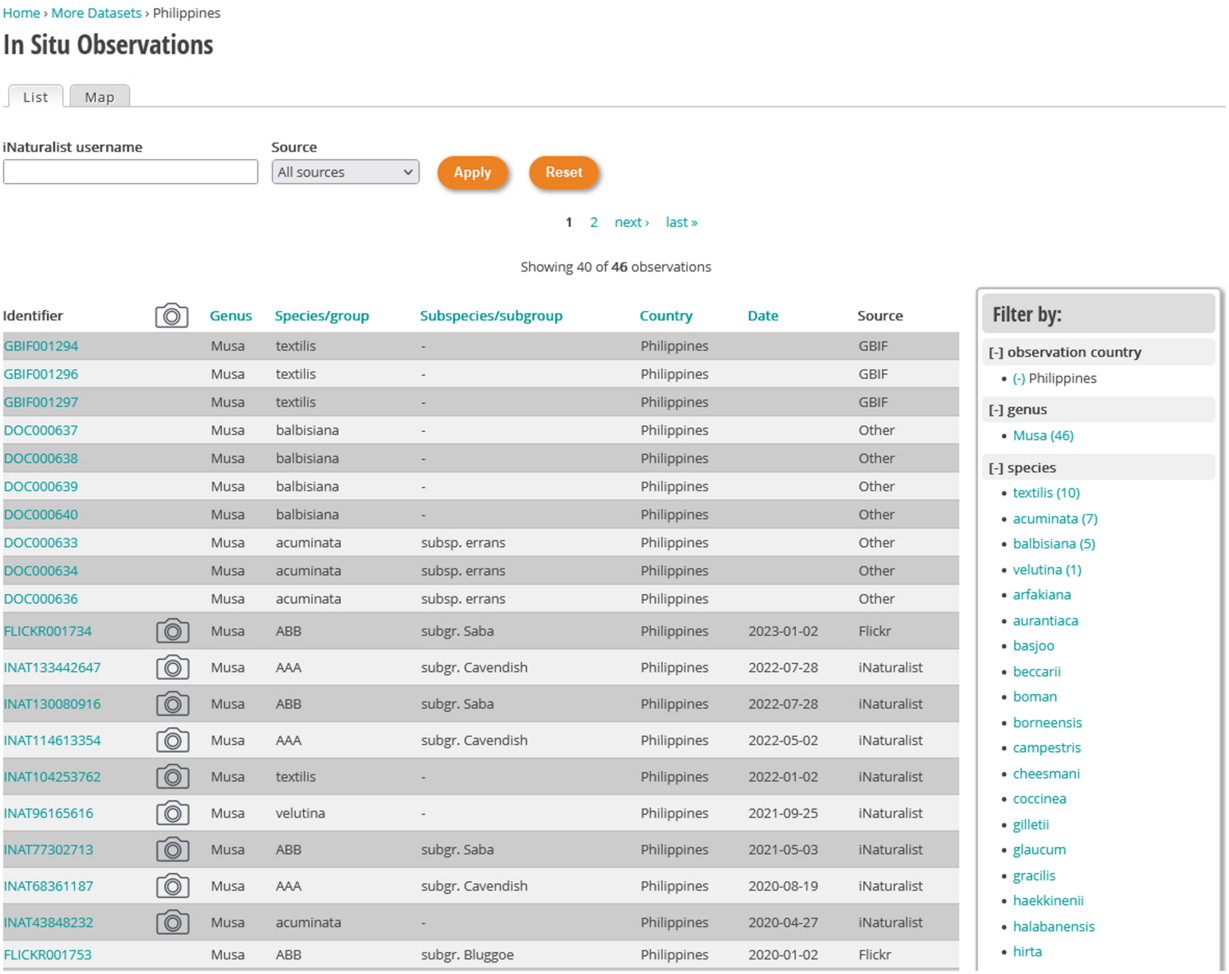
Examples of observations made in the Philippines obtained from various sources (publications, Flickr, iNaturalist) filtered with a facet search in MGIS.

**Figure 5.**
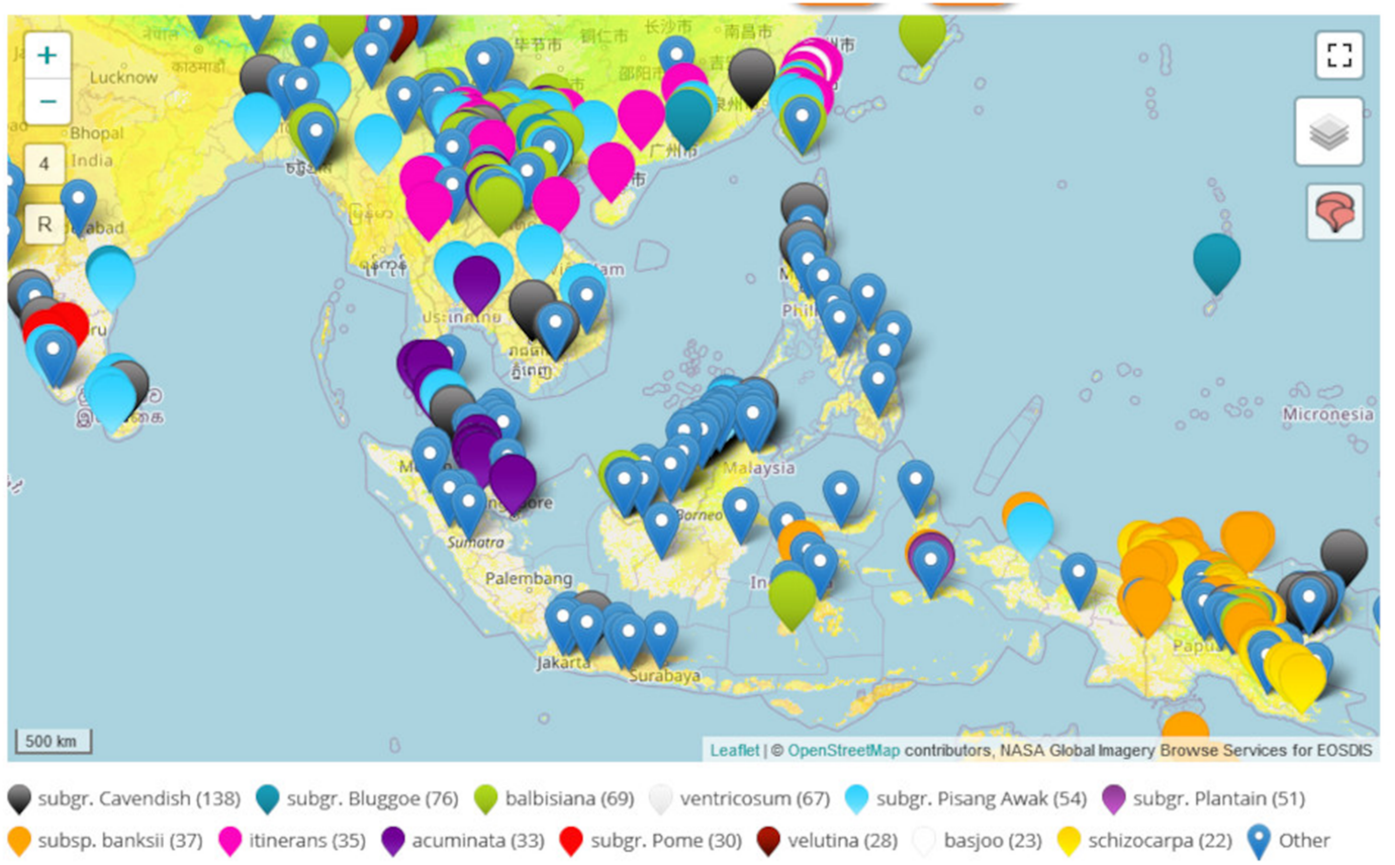
Map view of observations recorded in the center of origins of bananas in southeast Asia and Oceania. A color code indicates at glance the taxonomy of the observations and more details pop up by clicking on one of them (e.g. source, taxonomy, GPS location and photo).

The retrieval of observations can be performed in two ways: one way is a classical table view that is listing observations (Figure 4); the second way is a display of observations on a map (Figure 5). In both cases, a filter with multiple criteria can be applied to refine the search of observations. Three main criteria are available: country, taxonomy and observation date; for taxonomy, the taxon level goes from genus down to subspecies (wild) or subgroup (cultivated). It is also possible to select a data source such as iNaturalist, GBIF, Flickr, Pl@ntNet, Google Street View and Documents (include all technical and scientific literature). Once a filter is set, it applies to views, list and map. For registered users on iNaturalist, their own observations can be filtered and displayed within our tool by simply entering their username.

### Facet search

Filtering options are available on the right side of the page and allow to cross the three different criteria (taxonomy, country and date) to narrow down the number of observations (Figure 4). One of the key benefits of this facet search feature is its dynamic update mechanism. Each time a user selects a filter, the interface immediately updates to display the number of remaining observations between parentheses. All filters may be selected and combined. This real-time feedback assists users in making informed choices during their search process.

In the resulting table, every observation is assigned a unique identifier, which includes a prefix denoting the data source (for example, ‘INAT’ for observations from iNaturalist). An accompanying photo icon, when present, signifies that an image has been imported and linked to the respective observation. The table also provides a concise summary of each observation, encompassing essential details such as the genus, species or group, and subspecies or subgroups. Additionally, it includes the country and date of the observation, as well as the data source, offering a comprehensive snapshot of the recorded data.

### Map view

The page features an interactive map view, using OpenStreetMap, to visually represent observations in their recorded or collected locations. Additionally, users have the option to enrich the map with layers such as the land surface temperature layer, provided by NASA’s Global Imagery Browse Services (GIBS), as shown in Figure 5.

Interacting with the map is intuitive: dots on the map are color-coded to indicate the observation’s classification into groups, subgroups, species or subspecies. Clicking on a specific observation symbolized by a dot triggers a popup that provides detailed information about the observation, including photos when they are available. This popup also serves to access the full observation details. By default, observations are clustered to enhance map readability, with the number of clusters varying depending on the zoom level. For a more granular view, users can choose to unbox these observations. This is done by clicking the designated icon located at the top right corner of the map. To ensure a smooth and efficient page display experience, the map initially loads a maximum of 2000 observations. However, this limit is adjustable based on the filters applied by users.

### Observation view

Observation details are displayed on a specific page, the observation view. Its model and structure are based on the accession page, already existing for *ex situ* collections in MGIS and adapted to the subset of information for *in situ* observations. Morphotaxonomic descriptions and genomic analyses are not present since observations are based solely on an external photo and a more or less precise location.

**Figure 6.**
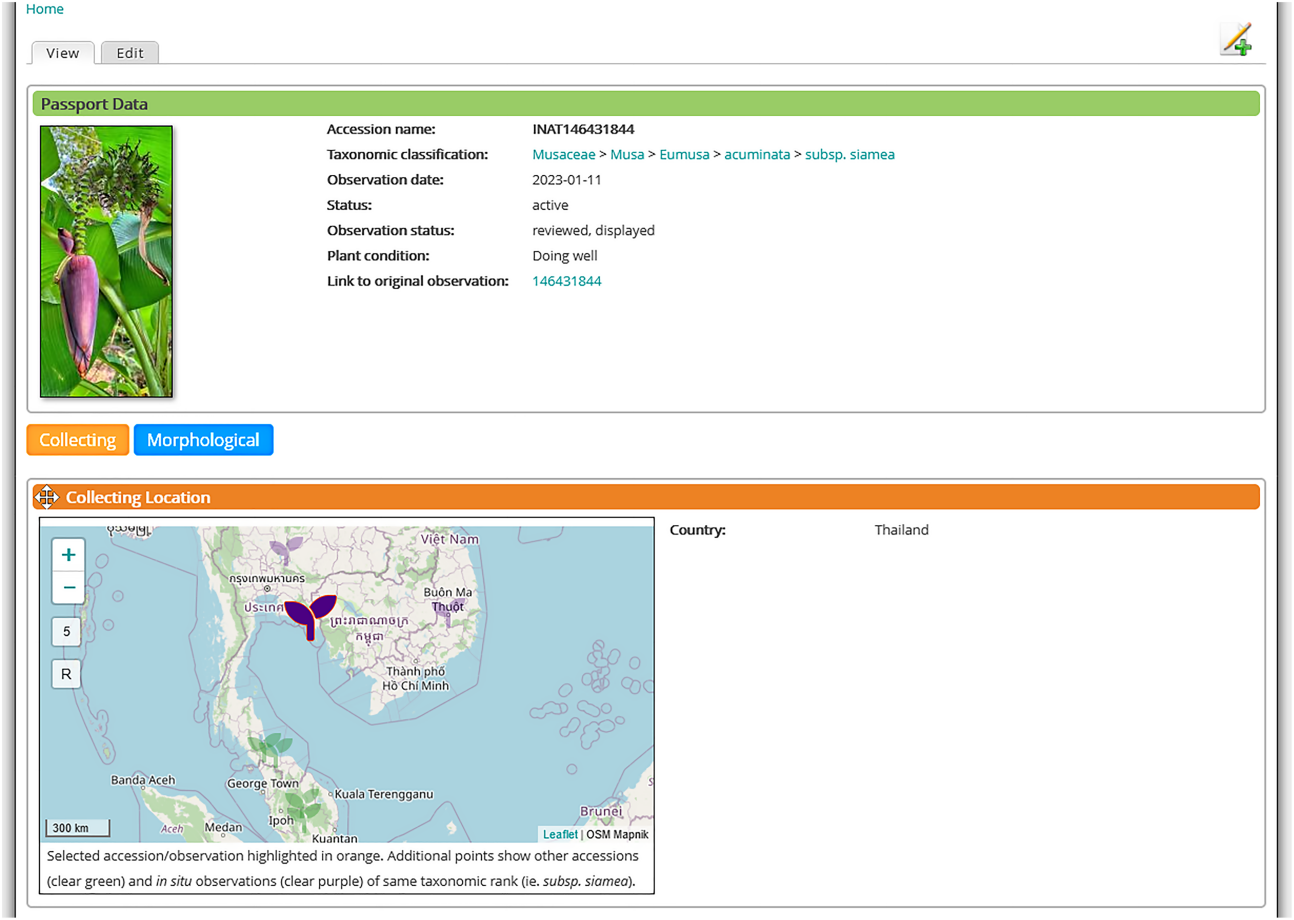
MGIS *in situ* observation of *Musa acuminata* subsp. *siamea* in Thailand (Icon with focus), surrounded by other *in situ* observations and *ex situ* accessions from collections collected in the same area, disciminated by a color code. Details on observation and accessions can be obtained by clicking on each icon.

The insertion of *in situ* data enabled the comparison of observed accessions and the origin of accessions maintained by *ex situ* collections (Figure 6). Both datasets are available within MGIS, which focuses primarily on passport data in *ex situ* collections. When the origin of an accession in a collection, or at least its place of collection, is documented, it is then possible to map it (in green in Figure 6), together with *in situ* observations of the same taxonomic rank (in purple in Figure 6). It is then easy, for example, to check whether an accession in a collection comes from a natural area where the species/subspecies is present. This is an invaluable information on the representation of natural diversity in *ex situ* collections and can highlight the need to collect new material in neighboring areas.

## Discussion

In the context of recording biodiversity, crowdsourcing is a cost-effective way to collect data, as it relies on voluntary remote help, without any need to set up expensive collecting missions and avoids tedious administrative constraints (e.g. obtaining permits, coordinating with local authorities or managing logistics). It also builds on local knowledge, thanks to the participation of native people from observation areas. From the inception of this project, and especially after launching the iNaturalist BaNaBioMap project, we have observed a spontaneous and steadily increasing participation from global users, who continue to propose new observations with increasing frequency. A still small but passionate network is making its way, and may lead to more numerous, accurate or otherwise difficult to reach observations, with possibilities for discussion and maybe even collaborative actions with material exchange for conservation and further characterization. In this regard, it deserves its citizen science labeling, building knowledge on both sides.

While crowdsourcing offers a wealth of benefits and has greatly enriched the MGIS database, it is important to note that it comes with its own set of known biases and limitations (5, 25, 26). First, one of the inherent biases is that the data collected often reflect the locations people frequently visit, or at least countries where mobile phones’ access and the internet are more common and/or easy. There is an overrepresentation of data from popular or easily accessible locations, while remote or less-popular areas are underrepresented. Aiming at mitigating the location bias, we explored ways to extend data sources with the explorative use of Google Street View (Figure 3) as reported also in other plants (27). As of November 2023, Africa is still, and by far, the least represented area, on systems, online databases and Google. Still, some countries are progressively covered, such as Ghana, Nigeria, Kenya, Tanzania, Madagascar, Uganda, as for countries of interest to our project. In our experience, these resources have brought insights only regarding the main known types, classified as Plantain, Cavendish, Red, Pisang Awak or Bluggoe for instance. This limitation is because only these types, which are commonly grown on road edges, are easy to distinguish, and do not necessarily need detailed botanical pictures to do so.

Secondly, an important question is whether there is confidence in the quality of the submitted information. On the one hand, contributors may have variable knowledge on the plants they observed, and on the other hand, online platforms are generic and do not specialize in any organism. In our case, this issue translated mostly into inaccurate taxonomic identification of plants. The disclaimers of all these databases indicate that they are neither specialized nor competent, and that they rely on the external expertise of data providers. No corrections are made once the data are included in the database. Consequently, for this project, we could not avoid the time-consuming manual step of data verification, which can only be carried out by a few experts around the world. Indeed, to date, no satisfactory automatic botanical identification tool has ever been proposed for bananas. Progress in image recognition using deep learning-based approaches will certainly help to streamline the curation process in the near future (28).

It is also noticeable that working on bananas, we benefit from the natural appeal of this big and popular plant, which is easy to spot, and remarkable for its stature, general aspect and common use where it is grown (29). A plant like this, which is widely recognized and liked, is more likely to generate abundant data compared with a lesser-known, smaller/less conspicuous or less popular plant species. Indeed, there is noticeable bias toward plants that are popular with the public: some species are more abundant, attractive and easier to photograph than others.

**Figure 7.**
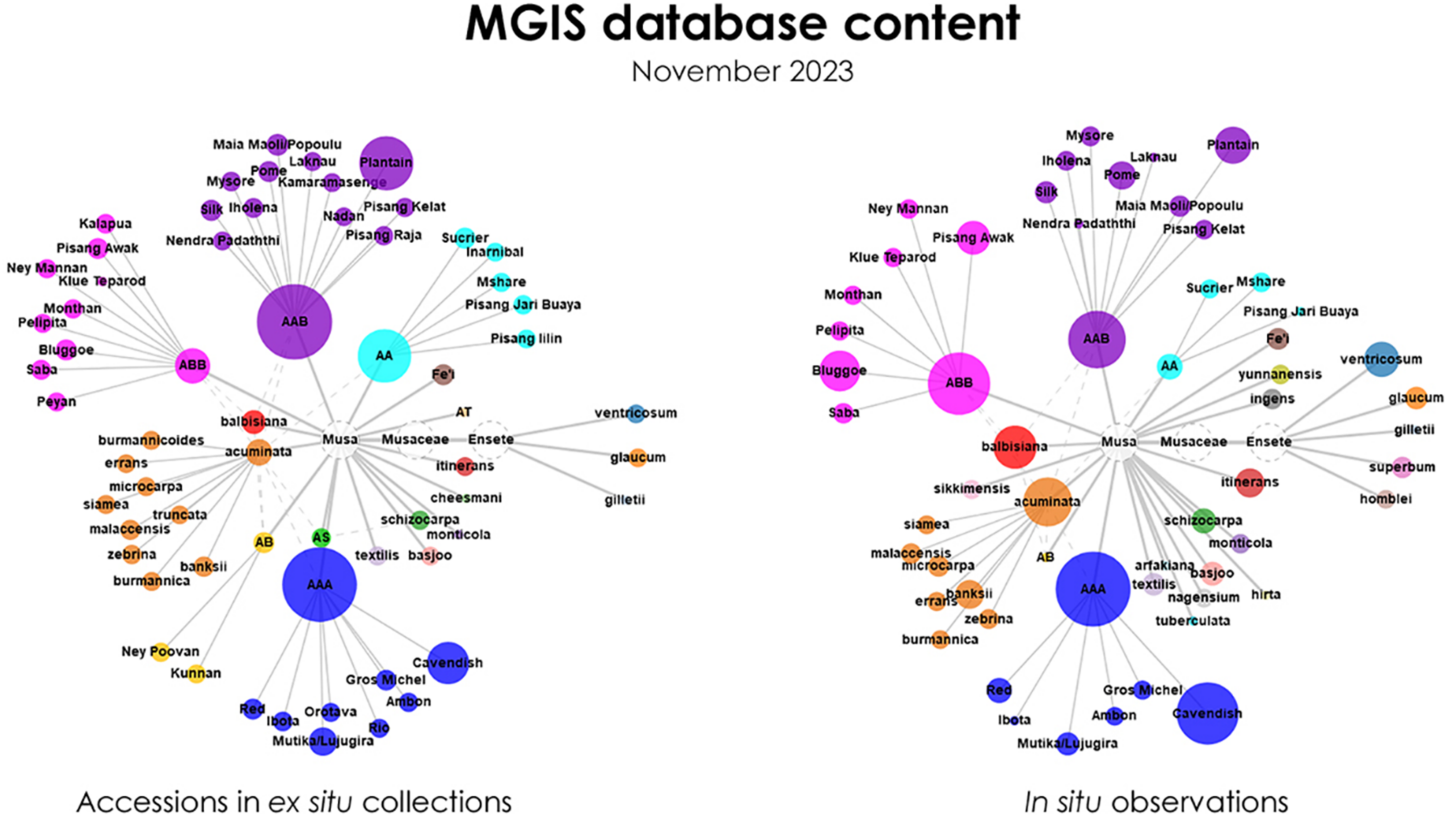
Diagram illustrating the *in situ* and *ex situ* taxonomic diversities of bananas, categorizing accessions and observations from family to subspecies/subgroup levels, as outlined by the MGIS. Bubble size corresponds to the quantity represented.

In conclusion, as the number of observations continues to grow, MGIS will continue to provide a global overview of banana’s natural diversity worldwide. This will be regularly enriched, more detailed and more precise, supporting analyses for relevant actions such as collecting missions for the conservation of banana genetic resources, or for breeding. The inclusion of *in situ* banana data in MGIS is a relevant choice for several reasons. Firstly, we benefited from the existing *Musa* taxonomic system to easily integrate and map the new information. For instance, the MGIS website homepage now displays and allows to compare the respective distributions of diversity *in ex* situ accessions and *in situ* observations included in the database (Figure 7).

Secondly, it successfully links *ex situ* and *in situ* data on genetic resources. This innovative feature enhances the thoughtful conservation of genetic resources, providing tools to predict the collection of endangered or missing resources. Because this is achieved using a citizen science approach, it also helps enable the necessary and effective network to be set up at the same time.

## Supplementary Material

baae036_Supp

## Data Availability

All data are available for free online viewing through the MGIS website at https://www.crop-diversity.org/mgis.
