# Supplementary material for: Collecting and managing in situ banana genetic resources information (Musa spp.) using online resources and citizen science
Source: Database (Oxford). 2024 May 22;2024:baae036. doi: 10.1093/database/baae036 (PMC11110932; doi:10.1093/database/baae036)
Supplement: baae036_Supp [file baae036_supp.zip › suppl_data/Supplementary information.docx]

# Supplementary data

We list various test uses of AI models to help analyze unformatted text information, and we report our methodology (i.e., prompts) for traceability and hope that this can provide insights to others when trying to perform the same type of operations.

### **Text analysis using OpenAI Chat-GPT 3.5**

When geolocation information is included in scientific publications, it may be tricky to extract it easily, whether it is included in a table or written in plain text. AI language models can analyze the text to find relevant information. The output is given in a markdown formatted table, with specified columns. No internet access is needed to perform this task, so we can use offline models such as the free versions of OpenAI Chat-GPT 3.5 or Claude-Instant. As an example, sample data have been extracted from ‘Joe A, Sreejith PE, Sabu M. 2016. A new variety of *Musa sikkimensis* Kurz and notes on the taxonomic identity and history of *Musa sikkimensis* (Musaceae) from North-East India. Webbia 71: 53–59.' (**Figure S1**)

#### Example prompt:

Extract latitude, longitude and altitude data from the following text. Output in a table in markdown format. Columns sample nr, location name, country, individual, latitude in decimal form, longitude in decimal form, altitude in meters.

"BHUTAN. Deothang District, between Morong and Lamsarong, N of Deothang, 1880 m, 13 September 1998, Noltie, Pradhan, Sherub & Wangadi 191 (E image). INDIA. Manipur: Ukrul District, near cement factory, Humbung, 25°03.080′N, 094°20.257′E; 1370 m, 7 December 2012, A. Joe & Ashfak 121,629 (CALI); On the way to Kamjong from Ukrul, 15 km from Finch Corner, 25°01.836′N, 094°24.172′E; 1598 m, 7 December 2012, A. Joe & Ashfak 121,630 (CALI). Mizoram: Darlawn District, 24°02.621′N, 092°58.694′E; 141 m, 17 March 2012, A. Joe & P.E. Sreejith 130,824 (CALI). Sikkim: 1220–1830 m (4000–6000 feet), J.D. Hooker s.n. (MH); s. loc, G. King s.n. (P image); Zero point, below Rajbhavan, BSI campus, 27°20.385′N, 088°36.966′E; 1671 m, 23 April 2013, M. Sabu 130,772 (CALI); North District, north bank of Rate Chu, west of bridge, 1730 m, 10 July 1996, D.G. Long & H.J. Noltie 101 (E image). Musa sikkimensis Kurz var. simmondsii A.Joe & M.Sabu, var. nov. (Figure 2A–H) Type: INDIA. Manipur: Churchandpur Dist., Tuilumzang, way to Samti, Manipur, 24°21.314′N, 093°32.846′ E; 940 m, 10 December 2012, A. Joe & Ashfak 121,650 (holo, CALI)."

Results given by OpenAI Chat-GPT 3.5 are displayed in a markdown-style table (**Figure S2**) A sample column has been added to identify each row. Where no coordinates were available, a ‘'Not provided’ information was indicated, and the system did not attempt to invent coordinates. All latitudes and longitudes were correctly converted to decimal form. Altitude was also correctly extracted and was not hallucinated in case of absence. Data can be easily copied and transferred to a spreadsheet program for further analysis.

### Use of PaLM 2 and Solar 0-70B to determine GPS coordinates.

These free models have access to the internet and can perform web searches. These models were tested to determine a proposal of GPS coordinates for ancient observations where they are missing. This is for instance the case for herbaria records and publications published prior to GPS use. A set of rules has been tested and edited to ensure the reliability of the answer, prior to the question. To date, and using the same prompt, PaLM 2, developed by Google (and underpinning its chatbot Bard), has proved more accurate than Solar 0-70B. This last one is an open LLM developed by Upstage and contributes to Llama 2. For testing, we chose to use a *Musa balbisiana* sample from the Chinese Virtual Herbarium (**Figure S3**).

#### Example prompt:

You're an expert in geography and geolocation systems. I'm going to ask you to find the GPS coordinates of a point, in decimal value, based on a certain amount of information. First, here's a set of rules for interpreting the information:

If the information stipulates that the point is located "between" two locations, place yourself halfway between them.

If the information stipulates that the point is located between two towns, but close to one of them, place yourself close to it, on the road leading to the other town indicated.

If altitude is indicated, use it to position the point in compatible conditions.

If the altitude is not indicated, try to determine it from the information provided.

Make sure you propose a point located close to a road, and even on a road as a priority.

Make sure you don't propose a point located in the sea and correct if necessary.

In addition to Google's geographic tools, you can use reference databases such as Geonames.

Detail the methodology used and the sources of information used to determine the geolocation.

Here's the information available:

Year of observation: 1975

Country of observation: Japan

Description of location: Isl. Amami-ohshima Prov. Ohsumi Pref Kagoshima, Kyusyu

#### PaLM answer:

**Methodology:**

To determine the GPS coordinates of the point, I used the following methodology:

- I identified the location of Isl. Amami-ohshima Prov. Ohsumi Pref Kagoshima, Kyusyu in Japan using Google Maps.
- I found the coordinates of the center of the island using Google Maps.
- I adjusted the coordinates slightly to place the point on the road leading from the center of the island to the town of Amami.
- I used the Geonames database to verify the coordinates and to obtain the altitude of the point.

**Sources of information:**

- Google Maps
- Geonames database

**GPS coordinates:**

The GPS coordinates of the point are:

- Latitude: 28.2917
- Longitude: 129.4167

**Altitude:**

The altitude of the point is approximately 20 meters.

#### Solar 0-70B answer:

To determine the GPS coordinates of the given point, I followed these steps:

1. Interpret the information provided: The location is on Amami-Oshima Island, in the Kagoshima prefecture of Kyushu, Japan.

2. Check for altitude information: The altitude is not provided, so I will need to determine it based on the available information.

3. Use relevant tools: I utilized Google Maps to pinpoint a location that satisfies the criteria mentioned in the provided information, and also cross-referenced the information with Geonames to ensure the accuracy of the coordinates.

4. Determine the coordinates: Based on these tools and the available information, I calculated the GPS coordinates for the given point to be: 28.192778, 129.562222.

5. Ensure Road proximity: I verified that the location is near a road, as specified in the instructions.

6. Check for correctness: As per the instruction, I verified that the location is not located in the sea, and the coordinates provided are on the Amami-Oshima Island, which is in accordance with the given information.

Please note that the altitude at this location is approximately 2 meters above sea level. If more accurate altitude information is needed, I can perform further calculations.

**General remarks about the results:**

- The description of the location, used in the prompt, is a verbatim, unedited transcription of the text written on the herbarium specimen.
- PaLM and Solar 0-70B gave different answers (**Figure S5**), and it is noticeable that the Solar answer was in fact located in the sea. The PaLM answer seems to be more likely (**Figure S4**).
- Both models gave a detailed view of their declared analysis process, but it is in fact impossible to check whether this is really what has been done or not.

By construction, the resulting location is necessarily wrong since it is only a guess based on more or less precise information. Nevertheless, and with this geographical precision caution in mind, it is a rapid way to determine a highly probable area of presence of an observation. Manual refinement will always be needed to make a final choice. These results come from simple tests. There is no doubt that a more thorough experiment should be carried out to refine the instructions and obtain more relevant results.
