# Supplementary figures and images for: Collecting and managing in situ banana genetic resources information (Musa spp.) using online resources and citizen science
Source: Database (Oxford). 2024 May 22;2024:baae036. doi: 10.1093/database/baae036 (PMC11110932; doi:10.1093/database/baae036)

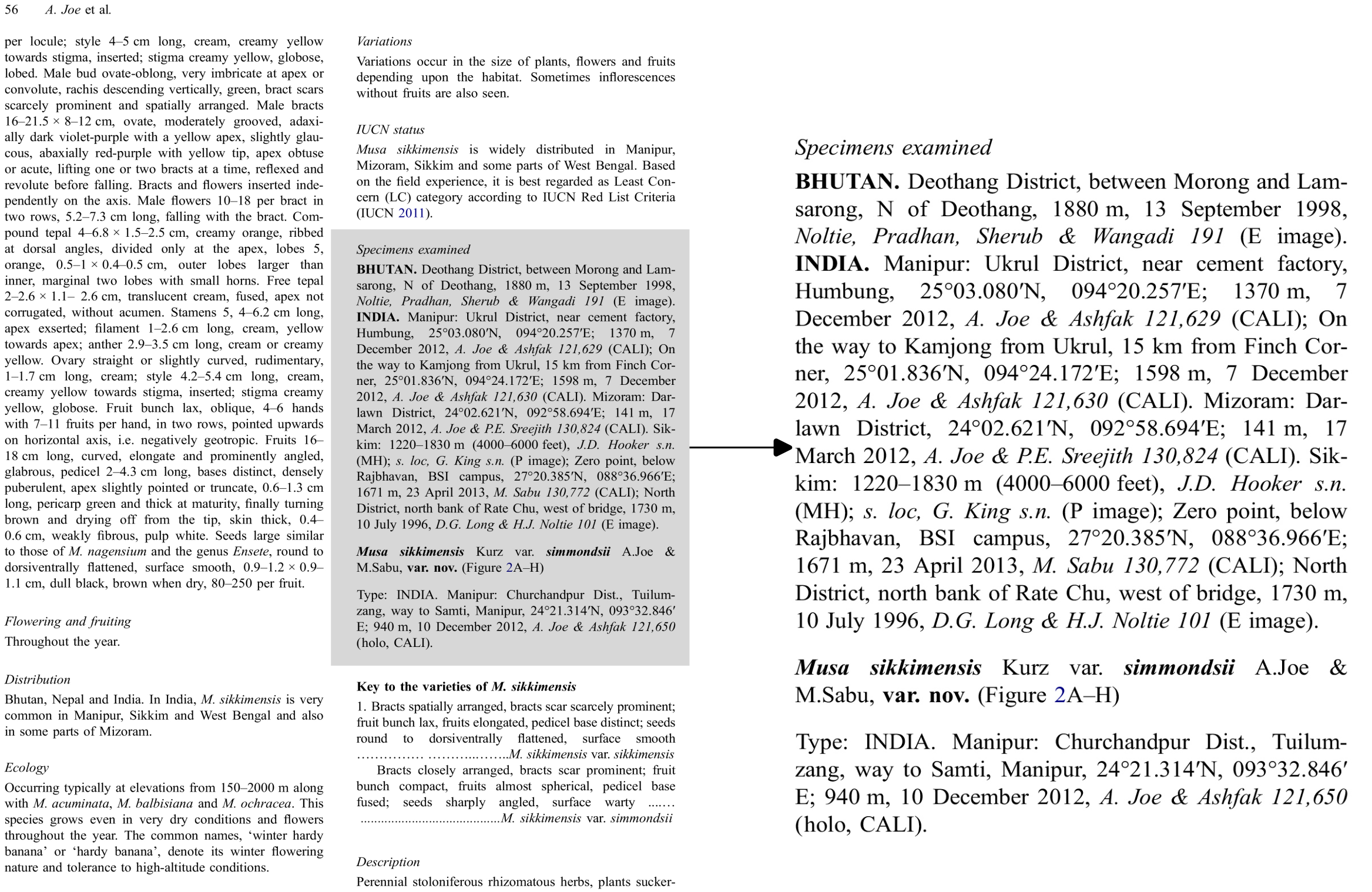

Supplement: baae036_Supp [file baae036_supp.zip › suppl_data/Figure_S1_300cpr_Sample publication for AI language model extraction text test .tif]

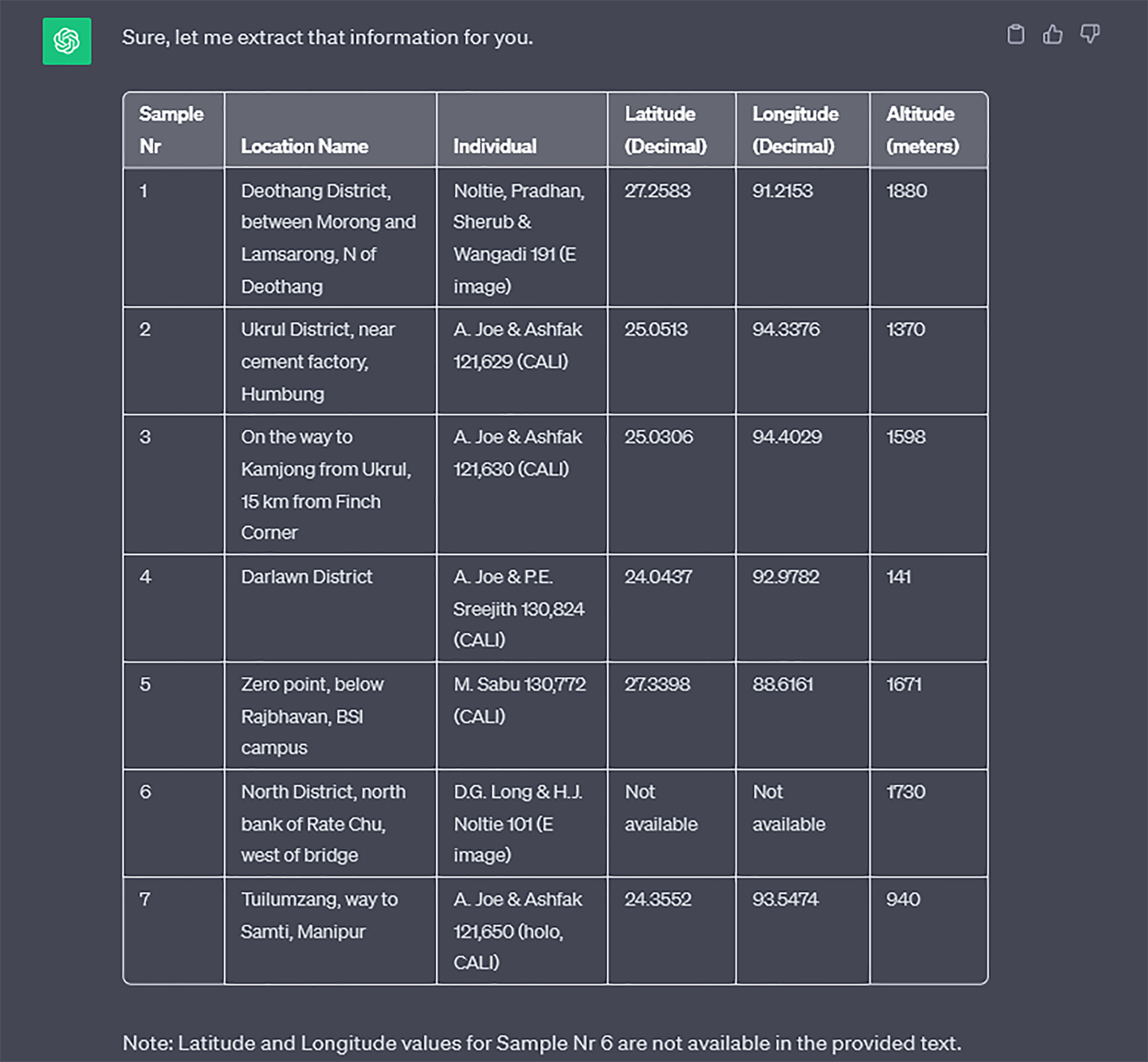

Supplement: baae036_Supp [file baae036_supp.zip › suppl_data/Figure_S2_300cpr_Markdown output of OpenAI Chat-GPT 3.5.tif]

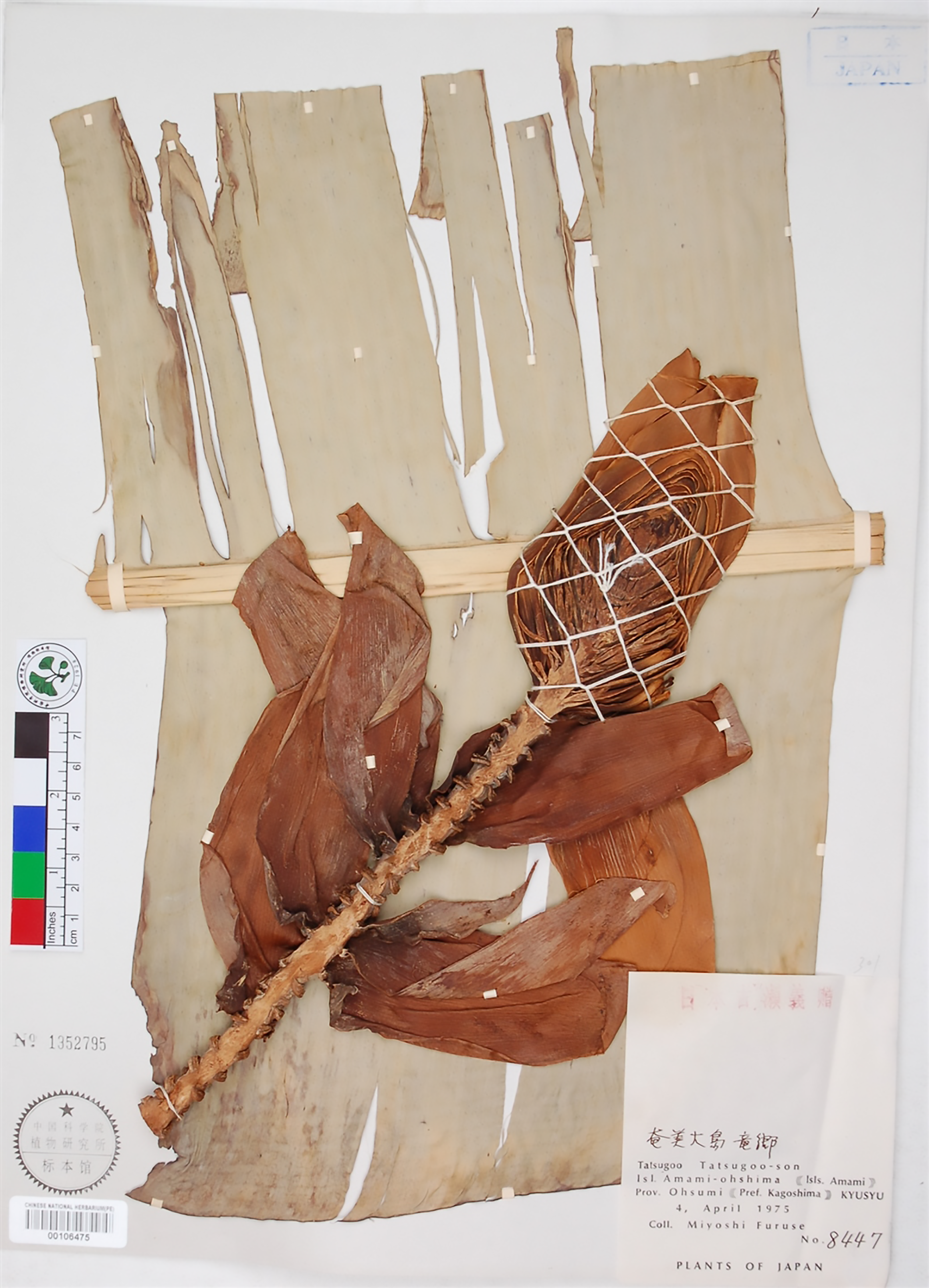

Supplement: baae036_Supp [file baae036_supp.zip › suppl_data/Figure_S3_300cpr_Herbarium sample of Musa balbisiana.tif]

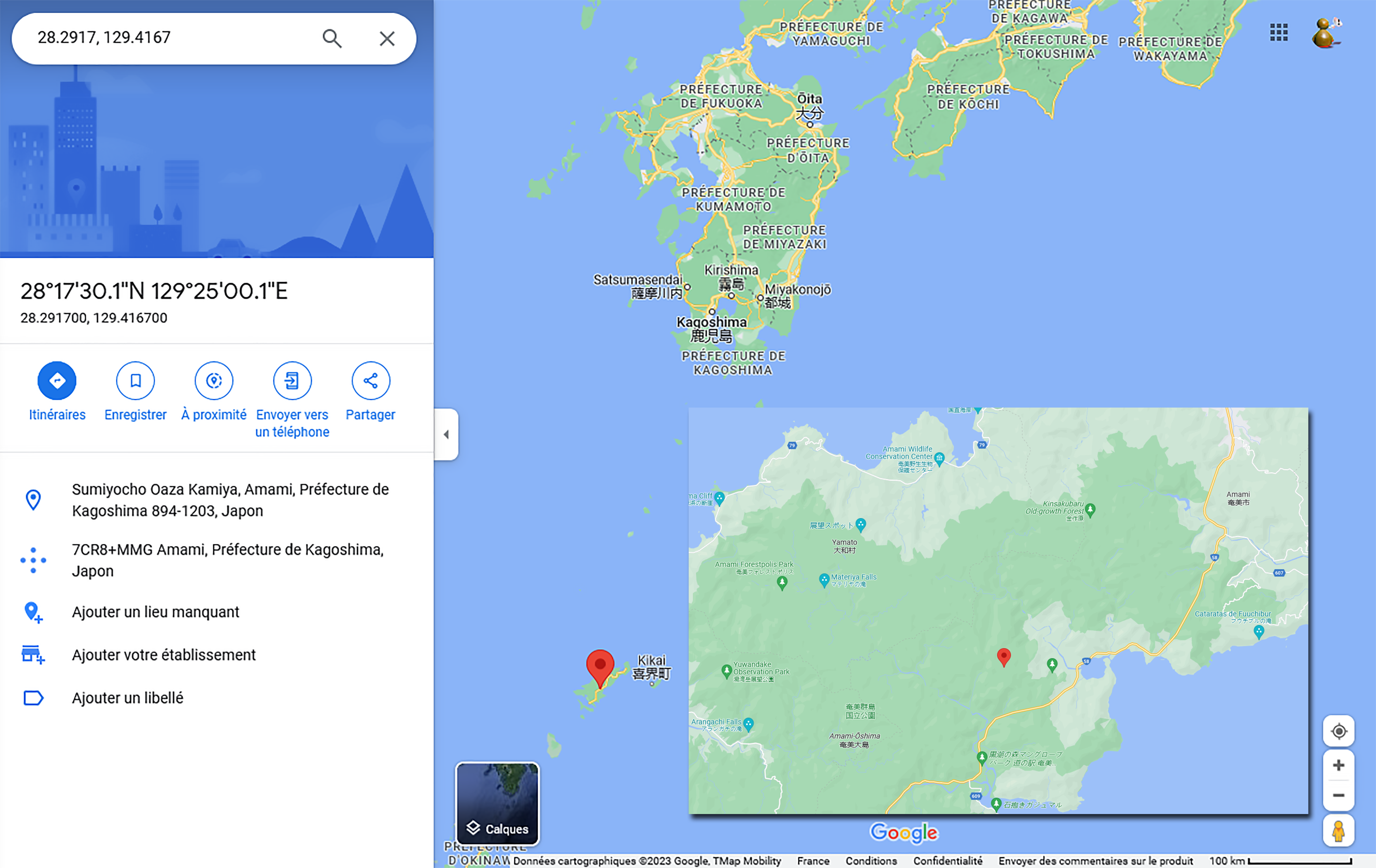

Supplement: baae036_Supp [file baae036_supp.zip › suppl_data/Figure_S4_300cpr_Location of the Musa sample.tif]

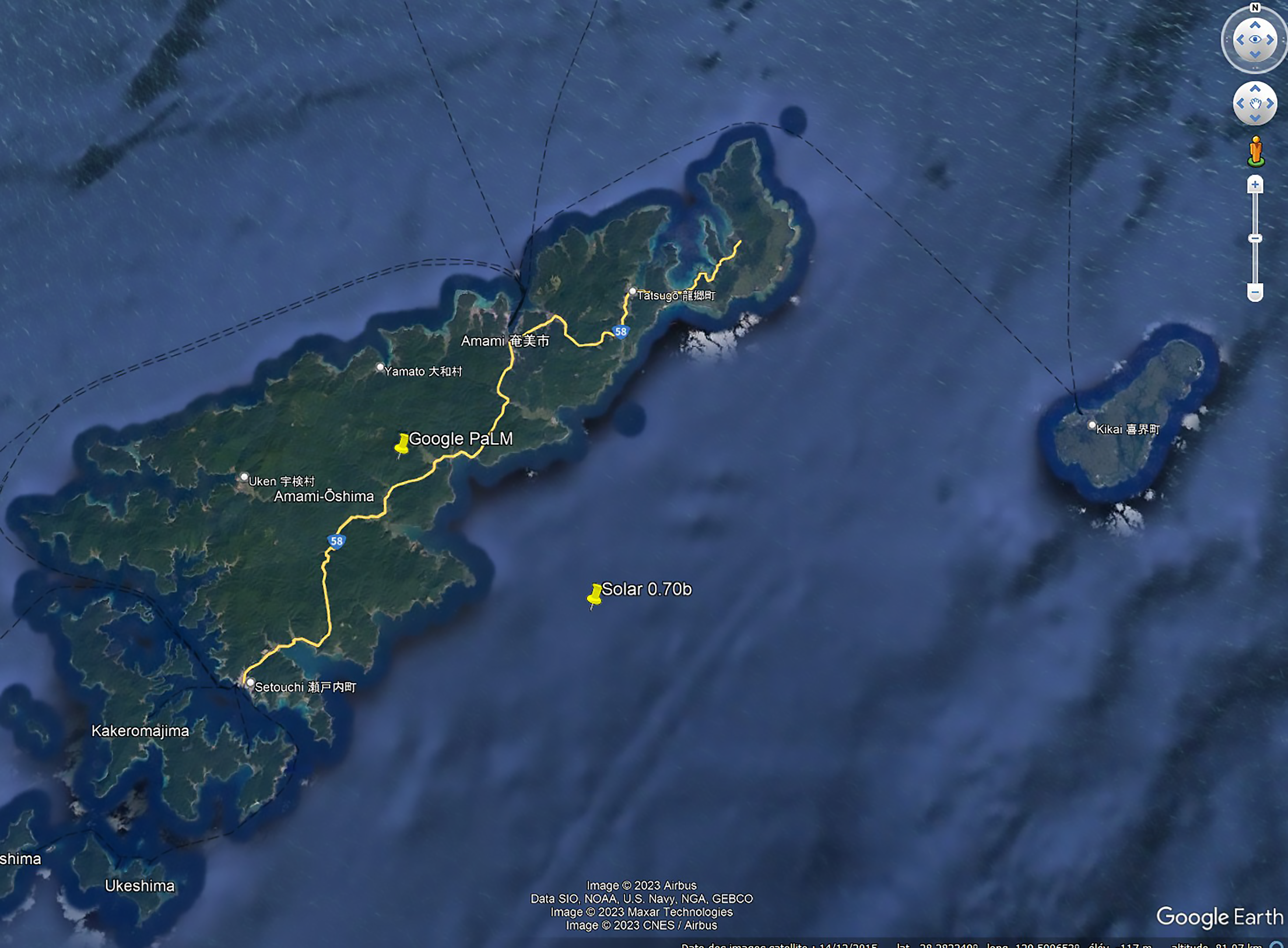

Supplement: baae036_Supp [file baae036_supp.zip › suppl_data/Figure_S5_300cpr_Positions of the herbarium observation, as determined by both AI language models.tif]
